# RECALL prompting hierarchy improves responsiveness for autistic children and children with language delay: a single-case design study

**DOI:** 10.3389/fpsyg.2024.1435688

**Published:** 2024-10-25

**Authors:** Rebekah Bosley, Susan J. Loveall, Karen Kate Kellum, Kara Hawthorne

**Affiliations:** ^1^Department of Speech and Hearing Science, University of Illinois at Urbana-Champaign, Champaign, IL, United States; ^2^Department of Communication Sciences and Disorders, University of Mississippi, Oxford, MS, United States; ^3^Department of Special Education and Communication Disorders, University of Nebraska – Lincoln, Lincoln, NE, United States; ^4^Department of Psychology, University of Mississippi, Oxford, MS, United States; ^5^Department of Hearing, Speech, and Language Sciences, Gallaudet University, Washington, DC, United States

**Keywords:** RECALL, dialogic reading, intervention strategies, pragmatic language, autism, language delay/disorder

## Abstract

The purpose of the current study was to expand upon previous research on RECALL, a dialogic reading intervention modified for autistic children aimed at increasing engagement. Children ages 3–6 years (*n* = 6) with language delays with or without co-occurring autism were tested using a multiple baseline across participants design. During baseline, the interventionist used dialogic reading and asked questions after every page. During intervention, the interventionist used RECALL, including a least to most prompting hierarchy with visual prompt cards. Children were more responsive and produced more meaningful correct responses during the intervention. Response type (linguistic vs. non-linguistic) also changed from baseline to intervention, though the pattern varied across participants. Intervention was not associated with increased responsiveness to adult bids for attention or pauses designed to encourage the child to initiate an interaction, though a few children showed changes in these responses over time.

## 1 Introduction

Parent–child shared book reading interventions, such as dialogic reading, can promote positive language outcomes in autistic children, including increased responsiveness, verbal participation, and book engagement (Alharbi et al., 2023; Boyle et al., 2019; Towson et al., 2021). RECALL (Reading to Engage Children with Autism in Language and Literacy) is a modified dialogic reading intervention aimed at improving engagement and responding for autistic children (Whalon et al., 2013, 2015). While promising, more converging evidence is needed to evaluate the efficacy of RECALL for autistic children. RECALL may also benefit other populations who struggle with social communication, including children whose primary diagnosis is a language delay/disorder, though this has not been previously examined. The purpose of the current study was to examine the effectiveness of the RECALL prompting hierarchy in improving pragmatic skills (responsiveness, meaningful correct responses, response types, attention, and initiations) in young children with language delays with or without co-occurring autism.

Autism is a neurodevelopmental disorder or difference characterized by difficulties in social communication – including pragmatics – and social interactions, as well as restricted and repetitive patterns of behavior, interests, or activities that are not otherwise explained by an intellectual disability (American Psychiatric Association, 2013). Most recent estimates are that one in 36 children in the United States is on the autism spectrum (Maenner et al., 2023). Pragmatic challenges include difficulties responding to comments or questions, expressing affirmations, providing expansions, negotiating verbal turn-taking, and producing contingent utterances (Casenhiser et al., 2015; Lam and Yeung, 2012; Reichow et al., 2008). Difficulties with joint attention are also common, relative to both typically developing peers (Bruinsma et al., 2004; Bottema-Beutel, 2016), peers with language delays (Loveland and Landry, 1986), and peers with intellectual and developmental disabilities (Mundy et al., 1990).

Approximately 3–8% of children in the United States have a language delay or disorder (Black et al., 2015; Norbury et al., 2016). These delays often include difficulties with pragmatics (Craig, 1993; Paul et al., 2008). For example, pragmatic difficulties in Developmental Language Disorder (DLD) include a decreased frequency of spontaneous communications and less sharing of emotions compared to typically developing children (Craig, 1993; National Institute on Deafness and Other Communication Disorders, 2022). Although children with DLD reportedly have stronger joint attention skills than their autistic peers (Loveland and Landry, 1986), they often still have difficulties relative to language-matched children without disabilities (Landry and Loveland, 1988). However, restricted and repetitive interests and behaviors are a core characteristic in autism not observed in children with language delays without co-occurring autism (American Psychiatric Association, 2013).

Dialogic reading is a reading intervention in which an adult works to actively engage a child in a storybook to improve language and literacy skills (Whitehurst et al., 1988). The adult engages the child via questions, prompts, and expansions. For example, two common dialogic reading strategies are the use of CROWD questions (*C*ompletion, *R*ecall, *O*pen-ended, *W*h-questions, and *D*istancing) and the PEER prompting hierarchy. With PEER, the adult *prompts* the child with a question (e.g., “What did the dog do in the yard?”), then internally *evaluates* and *expands* on the child’s response (e.g., “Yes, the dog runs!”). Finally, the child is taught to *repeat* the expanded response (Lonigan and Whitehurst, 1998; Whitehurst et al., 1988, 1994).

There is a large body of literature demonstrating that dialogic reading promotes language and literacy development, such as improvements in attention, reading attitudes, print awareness, alphabet knowledge, phonological awareness, and receptive and expressive vocabulary (for reviews, see: Dowdall et al., 2020; Mol et al., 2008; Pillinger and Vardy, 2022; Towson et al., 2017, 2021; Works Clearinghouse, 2007, 2010). These benefits have been documented across a wide range of populations, including typically developing children (Kotaman, 2020), children from low socio-economic status households (Vally et al., 2015), dual language learners (Huennekens and Xu, 2016), deaf and hard of hearing children (Fung et al., 2005), children with language delays/disorders (Crain-Thoreson and Dale, 1999; Crowe et al., 2000; Dale et al., 1996; Desmarais et al., 2013; Ramsey et al., 2021), children with intellectual and developmental disabilities (Jeremic et al., 2023; Towson et al., 2016), and autistic children (Fleury et al., 2014; Grygas Coogle et al., 2018).

Given their difficulties in social communication, autistic children and children with language delays/disorders without co-occurring autism may also benefit from dialogic reading techniques adapted to include a focus on pragmatic skills. Previous research has indicated that the use of least to most prompting (i.e., prompts that offer increasing assistance), visual cues, adult scaffolding, modified text, and tactile objects during shared-book reading are beneficial in promoting several pragmatic language outcomes for autistic children, including storybook engagement, responding to adult questions, spontaneous language use, and joint attention (Bellon et al., 2000; Grygas Coogle et al., 2018; Fleury and Schwartz, 2017; Mucchetti, 2013; Müller et al., 2023; Patten and Watson, 2011; Wong et al., 2015). For example, Fleury and Schwartz (2017) reported improvements in engagement and responsiveness in nine autistic children ages 3;0–5;11 during an intervention involving modified dialogic reading with least to most prompting. Although similar interventions would likely be useful for children with language delays/disorders without co-occurring autism, there has been limited research on using modified dialogic reading interventions with this population.

RECALL is an adapted version of dialogic reading designed to target pragmatic skills for autistic children (Whalon et al., 2013, 2015). RECALL includes a modified version of the PEER prompting sequence called PEEP, in which the adult concludes each sequence by *praising* the child rather than having the child *repeat* the adult’s expansion. RECALL also includes dialogic reading-type CROWD questions, as well as two additional question types: *wh-inference*, to help participants make inferences about what might happen next in the story, and *emotion identification*, to help participants identify the emotions of the characters. Additionally, RECALL includes *a visual least to most prompting hierarchy* to scaffold responding, with sequentially fewer visual response options following each incorrect response (See Procedures I). RECALL also encourages active participation and spontaneous language use from the participants via (1) *secure attention prompts,* in which the adult points to an object or action in the book, exclaims “Wow!” or “Look!,” and waits expectantly for the child to respond, (2) *intentional pauses*, in which the adult pauses and looks expectantly at the child for 3–5 seconds to see if they will initiate an interaction, and (3) *initiation question cards*, in which the child is cued to ask their reading partner a question about the story (See Procedures II for a more detailed description). Peers can also serve as interactive reading partners and social models during the interventions.

In their original study with four 4-5-year-old autistic males, whose communication skills included signing, echolalia, one to two-word utterances, and sentences, Whalon et al. (2015) reported that RECALL was effective at increasing participants’ responsiveness (i.e., the frequency of incorrect or no responses decreased) and spontaneous or “unprompted” correct responses. Three participants also increased their frequency of spontaneous initiations across the study, though data regarding responses to secure attention prompts, intentional pauses, and initiation question cards were not reported. Further, each participant was paired with a peer who was able to participate in the intervention by both asking and answering questions, though no data were presented on the peers’ involvement.

Subsequent studies have also suggested that RECALL is effective at improving engagement and communication outcomes for autistic children. For example, Nunes et al. (2022) used an ABAB design to compare RECALL versus another dialogic reading intervention with eight 3–5 year old children (four autistic children and four non-autistic peers matched on mental-age) and reported that RECALL led to greater vocabulary gains for participants. Further, a randomized control trial of RECALL by Lo and Shum (2021) found that 6 weeks of RECALL intervention, as implemented by parents who received training, improved reading engagement, responsiveness, story comprehension, and emotion knowledge in a sample of autistic preschoolers (ages 3–6 years) when compared to a control condition in which parents did not receive training. Thus, RECALL seems to be a promising intervention for young autistic children, though additional research is needed to strengthen its evidence-base, including how it compares to other dialogic reading strategies.

Given its highly structured format, use of visual prompt cards, and increased opportunities for responding, RECALL may also be effective for children with language delays/disorders without co-occurring autism. Dialogic reading strategies, broadly, have been effective at improving language skills in children with language delays/disorders (Crain-Thoreson and Dale, 1999; Dale et al., 1996; Ramsey et al., 2021; Whitehurst et al., 1994). However, Dale et al. (1996) indicated that more intensive interventions may promote more effective outcomes for children with language delays/disorders. For example, the added structure and increased opportunities to respond within the RECALL intervention may lead to greater engagement, learning, and language development for this population.

Therefore, the current study investigated the impact of the RECALL prompting hierarchy on communicative responses by replicating Whalon et al.'s (2015) single-case design study (1) with a larger age range (3–6 years vs. 4–5 years), (2) with a larger sample size (6 vs. 4 participants), (3) in children with language delays with and without an autism diagnosis, (4) and including data on variables not reported by Whalon et al. (2015): prompting level and response to adult bids. The baseline was dialogic reading with questions after each page, as well as secure attention prompts and intentional pauses; the intervention added the RECALL visual prompting hierarchy.

The research questions for this study were as follows: (1) Is there a functional relation between the intervention (i.e., RECALL visual prompting hierarchy) and children’s responsiveness when asked a story-related question? (2) Is there a functional relation between the intervention and the children’s rate of responses, including initial responses, defined as correct or incorrect responses, and meaningful responses, defined as correct responses that are given when there is more than one response option to choose from? (3) Is there a functional relation between the intervention and the children’s types of responses: linguistic (spoken or signed words or phrases) versus non-linguistic (gestures or pointing)? (4) Is there a functional relation between the intervention and the level of prompting required over time? (5) Do children’s responsiveness to adults’ secure attention prompts and intentional pauses increase over time?

## 2 Materials and methods

The Single-Case Reporting guideline In BEhavioral intervention (SCRIBE) 2016 checklist (Tate et al., 2016) was used to guide methods and results reporting.

### 2.1 Participants

Participants were six 3-6-year-old children (five male, one female; all Caucasian) with language delays (Table 1). Participants were recruited from a university-based preschool laboratory program for children with moderate-to-severe language delays. All children in the program were invited and agreed to participate in the study. Two participants had a diagnosis of language delay only (one moderate and one severe), and six participants had a diagnosis of autism + language delay. Participants’ primary modes of communication ranged from gestures to multi-word utterances, as reported by their speech-language clinician. This study was approved by the university’s Institutional Research Board; parents and participants provided consent and assent, respectively.

**Table 1:** Participant demographics and assigned intervention groups.

				Language						
Name	Age	Diagnosis	IQ	Auditory Comprehension	Expressive Communication	Total Language	Vocabulary	Primary Mode of Communication	Dyad	Interventionist
Dillan	5;1	A + LD	47‡	50*	50*	50*	56	Gestures	MBL (1)	2
Hayley	6;10	A + LD	92‡	67*	50*	55*	75	2+ word utterances; echolalia	MBL (1)	1
Ben	3;7	Moderate LD	54*‡	53*	72*	60*	51*	1-4-word utterances	MBL (2)	2
Tucker	4;10	Severe LD	57‡	51*	54*	50*	56	1-2-word utterances; gestures	MBL (2)	1
Oliver	3;2	A + LD	**†	55*	50*	50*	**	Gestures; non-linguistic vocalizations	MBL (3)	2
Wally	3;3	A + LD	**†	50*	50*	50*	**	Gestures; non-linguistic vocalizations	MBL (3)	1

*Age* is age at the start of the study. All reported scores are standard scores. Scores marked with * were obtained 7–8 months after the study; all others were obtained 0–4 months before. When an assessment was not scorable, it is marked with **. *IQ* scores marked with ‡ are the Kaufman Brief Intelligence Test - Second Edition (Kaufman and Kaufman, 2004) and those marked with † are T-scores from the Mullen Scales of Early Learning (Mullen, 1995); the Mullen was used when participants did not meet the minimum age for the KBIT-2. *Language* scores are from the Preschool Language Scales - Fifth Edition (Zimmerman et al., 2011). *Vocabulary* scores are from the Peabody Picture Vocabulary Test - Fourth Edition (Dunn and Dunn, 2007). *Primary Mode of Communication* indicates what the child’s regular graduate clinician reported as their typical responses. *Dyad* indicates pairs of participants in the MBL design (see Design). A = autism. LD = Language delay.

### 2.2 Interventionists, setting, and timeline

Each participant was randomly assigned to have the intervention administered by either the first author or a research assistant. The interventionists were both speech-language pathology students with prior research experience. Both interventionists trained on the same procedures and used the same materials, processes, and techniques. For Oliver, Wally, Ben, and occasionally Hayley, a graduate student clinician who was familiar with that child was present in the room to assist with behavior management. These graduate student clinicians were completing a clinical placement within the preschool program, and they worked one-on-one with the child each day as part of their regular therapy. Graduate student clinicians were instructed not to prompt or respond for the participant.

The study took place in small rooms used for therapy at the children’s on-campus preschool. The preschool was a morning-only program that involved daily individual and group therapy sessions, as well as circle time, book time, and other traditional preschool activities.

The study took place during a 6-week summer semester over 17–22 sessions of 20 min each. There were four sessions per week during Weeks 1–3 and 5–6. Due to a national holiday, there was a week-long break before Week 4, which had only two sessions. Each week, each child was asked to select between two storybooks and their selected book was read for that entire week. Because Week 4 was short, the same book was used for Weeks 4 and 5.

### 2.3 Procedures I: assessing responsiveness, accuracy, response type, and prompting level

Baseline and intervention procedures were adapted from Whalon et al. (2015). During both phases, the interventionist used the PEEP sequence after reading each page (see Whalon et al., 2015). First, they *prompted* for a response by asking an initial question. The questions included both traditional CROWD-type questions, as well as RECALL-specific wh-inference and emotion-identification questions (see Materials, below). Then the interventionist *evaluated* the response as correct or incorrect. If a participant provided a correct answer, the interventionist *expanded* upon the child’s response by adding additional vocabulary and *praised* the child for their correct response. For example, if the child responded to the *prompt,* “What did the dog do in the yard?” by saying or pointing to a picture of “run,” the interventionist would internally *evaluate* the response as correct and verbally *expand* upon the answer with, “Yes, you are right! The dog ran fast through the grass in the yard.” The interventionist would then *praise* the child, “Great job telling me what the dog did!”

Baseline and intervention differed in how incorrect responses and non-responses were handled after the initial question. In baseline, the interventionist provided the correct response and continued with the PEEP sequence by *expanding* on the child’s response and encouraging (*praising*) the child to keep participating (e.g., “Let us keep reading!”). In intervention, the interventionist proceeded through the RECALL visual least to most prompting hierarchy, described below.

#### 2.3.1 Intervention: RECALL prompting hierarchy

In intervention, an incorrect or non-response to the initial question (i.e., **Level 0** prompt) was followed by the RECALL visual least to most prompting hierarchy. Presence or absence of this prompting hierarchy constituted the independent variable.

**Figure 1:**
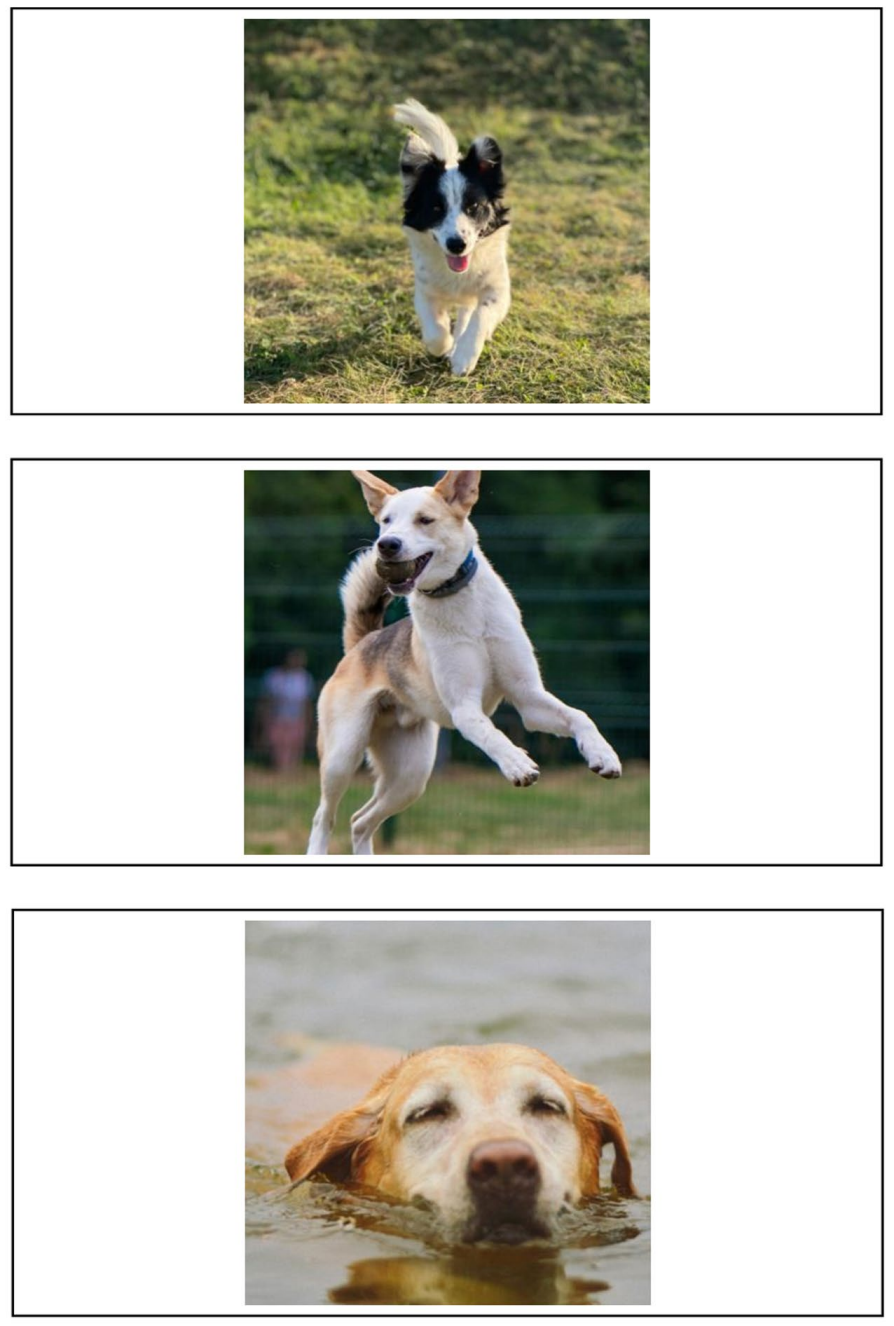
Example visual prompt cards. Visual prompt cards for the question, “Did the dog run, jump, or swim?” from *Dog’s Colorful Day*. Images obtained from Unsplash.

- *Level 1*: the interventionist repeated the question, then laid out three visual prompt cards as response options (e.g., photos of a dog running, swimming, and jumping; see Figure 1), while verbally listing the options (e.g., “Did the dog run, jump, or swim?”). If the child did not respond after ten seconds or responded incorrectly, the interventionist removed one of the incorrect prompt cards. For example, if the child incorrectly chose the picture of the dog swimming, that card was removed and the interventionist said, “No, the dog did not swim in the yard.” The interventionist then moved to a Level 2 prompt.
- *Level 2*: the interventionist repeated the question and pointed to the two remaining prompt cards while verbally naming them (e.g., “Run or jump?”). If the child did not respond after ten seconds or responded incorrectly, the interventionist removed the final incorrect prompt card. For example, if the child incorrectly chose the picture of the dog jumping, that card was removed, and the interventionist said, “No, the dog did not jump in the yard.” The interventionist then moved to a Level 3 prompt.
- *Level 3*: the interventionist repeated the question, produced the correct answer (e.g., “The dog runs in the yard.”), and repeated the question a second time with only the final, correct prompt card shown. If the child did not respond after ten seconds or responded incorrectly, the interventionist moved to a Level 4 prompt.
- *Level 4*: the interventionist repeated the question, then provided a hand-over-hand response for the child by moving the child’s hand to point to the correct prompt card while repeating the correct response (e.g., “The dog runs in the yard.”). The interventionist then removed her hand and asked the question a final time. If the child failed to provide an independent response at this point, it was coded as a no response.

A variety of response options were accepted throughout the prompting hierarchy, including speech, signing, and gesturing/pointing.

### 2.4 Procedures II: assessing responsiveness to adult bids

In addition, baseline and intervention both included three secure attention prompts and three intentional pauses per session. For secure attention prompts, the interventionist exclaimed in an exaggerated voice, “Look! ___!” (e.g., “Look! A dog!”) and pointed to a character, object, or action in the book. The interventionist then looked to the child, looked back to the picture, and waited for ten seconds to determine if the child would respond by looking and/or pointing at the character, object, or action. For intentional pauses, the interventionist looked expectantly at the page, then at the child, back to the page, and finally back to the child within a five second timeframe to determine if the child would initiate verbal or nonverbal communication. We chose to include these bids in both baseline and intervention, unlike Whalon et al. (2015), because it otherwise would not have been possible to compare responses to bids across phases. Further, we did not predict rapid improvement in responding to either type of bid, so this allowed us to examine if there was improvement over time.

Unlike Whalon et al. (2015), initiation question cards, in which the child is cued to ask a peer reading partner a question about the story, were not included because we did not include peers in the intervention.

### 2.5 Materials

Materials included storybooks from the *Read Together, Talk Together Kit A* from Pearson Education, Inc. (see Supplementary material S1), as well as session tracking sheets and visual prompt cards. The remaining materials were created specifically for the intervention. The research team established validity of the materials by examining books, questions, and visual prompt cards to determine clarity and appropriateness.

Each session had its own tracking sheet, which included CROWD questions and wh-inference and emotion identification questions (Table 2; see Supplementary material S1 for a complete example of questions from one session). The latter two question types are specific to RECALL and are intended to help children apply situations from the book to everyday occurrences and recognize emotions displayed by the characters, respectively (Whalon et al., 2013). The number of trials ranged from 12 to 14 per session, depending on the number of pages in the book. There were two of each question type when there were sufficient pages; otherwise, the number of each type of question was balanced across sessions and books.

**Table 2:** Question types (Whalon et al., 2013).

Question Type	Description	Example
Completion	A pause left the end of a sentence in place of a predictable word	*Finish what I say, “Now Dog has ten…”*
Recall	Asking what happened in the story	*Where did Dog run to?*
Open-Ended	Asking what is happening in the story	*Tell me what you see on this page.*
Wh-Questions	Asking wh-questions related to book vocabulary	*What is Dog holding?*
Distancing	Asking the child to relate the book to their personal experience	*What type of ice cream do you like best?*
Wh-Inference	Asking wh-questions that require prediction or understanding a character’s motivation	*What do you think Dog will do next?*
Emotion Identification	Asking how a character is feeling or how the participant would feel in a similar situation	*How do you think Dog feels?*

Although the same book was used every day for a week, the interventionists asked different questions each day to prevent memorization of the questions and answers. In addition, each session tracking sheet included three notifications to the interventionist to initiate a secure attention prompt and three notifications to initiate intentional pauses at either the beginning or end of a page. Interventionists also used the tracking sheets to collect data on the children’s responses.

Each intervention trial included three visual prompt cards (e.g., Figure 1) that were used during intervention only. Each trial’s cards involved either all photographs or all clipart images (e.g., three separate photographs of a dog running, jumping, and swimming). All images had clean backgrounds, with the focus on a single image of an object, character, or action.

### 2.6 Design

Participants were tested using a multiple baseline across participants (MBL) design. MBL designs allow for the detection of longer-term impacts of repeated intervention and can be used if researchers suspect the effects of intervention cannot be withdrawn (Kazdin, 2011). Given the short time frame of the intervention, the remaining six participants were randomly yoked into three dyads for the purpose of deciding when to move participants from baseline to intervention. However, all participants received the intervention individually. To decide when to move dyads from baseline to intervention, the researchers monitored the percent of initial correct responses, following Whalon et al. (2015) (see Variables and Analysis below, Research Question 2). The first dyad (Dillan and Hayley) moved to intervention after both showed a stable baseline for two sessions. Subsequent dyads moved to intervention once the earlier dyad was consistent in their number of initial correct responses in intervention for at least two sessions. Dyads 1, 2, and 3 began intervention in sessions 6, 9 (Ben)/10 (Tucker), and 15 (Oliver and Wally), respectively. Ben’s move to intervention was one session early due to interventionist error. To examine if we could quickly see effects of the intervention, two additional participants were tested using an alternating treatments design. Information about these participants and their results are reported in Supplementary material S2.

### 2.7 Variables

The independent variable was the method of reading: baseline (dialogic reading followed by the PEEP sequence on each page, as well as joint attention and intentional pause bids) versus intervention (baseline plus the RECALL least to most prompting hierarchy with visual prompt cards).

The dependent variables are listed below:

- Research question 1: *Responsiveness*

- *Percentage of initial responses*: For each trial, the initial response to a Level 0 prompt (i.e., an initial question with no visual prompt cards) was coded as *yes* if the child responded (correctly or incorrectly) or *no* if the child did not respond. All baseline responses were initial responses.
- *Percentage of overall responses*: For each trial, the overall response was coded as *yes* if the child responded (correctly or incorrectly) to a Level 0–4 prompt and *no* if the child failed to respond to all prompts. Initial and overall responses are equivalent in baseline since there was only one opportunity to respond.

- Research question 2*: Response Accuracy*

- *Percentage of initial correct responses.* For each trial, the initial response was coded as *yes* if the participant responded correctly to a Level 0 prompt and *no* if the participant responded incorrectly or did not respond at Level 0. All correct baseline responses were initial correct responses. Note that this variable was used to determine when MBL participants moved to intervention, following Whalon et al. (2015).
- *Percentage of meaningful correct responses*. For each trial, responses were coded as *yes* (i.e., *meaningful and correct*) if the participant responded correctly during Levels 0–2. At these prompting levels, a correct response demonstrates basic comprehension because the child is selecting between at least two prompt cards. A response was coded *no* (i.e., *non-meaningful*) if it occurred during Levels 3–4 because at this point there was only one remaining response option. If the child did not respond, it was also coded as *no.* Initial correct and meaningful correct responses are equivalent for baseline because all responses were after a Level 0 prompt.

- Research question 3*: Response type percentage*

- *Response type percentage*. Each participant’s final response for each trial was coded as linguistic, non-linguistic, combination, or no response. *Linguistic* responses included words (i.e., one-word utterances either spoken or signed) and phrases (i.e., multi-word utterances). *Non-linguistic* responses included gestures (e.g., head nods or head shakes) and pointing (e.g., with a single finger, multiple fingers, the whole hand/fist, and/or by picking up and handing a visual prompt card). *Combination* responses included both a linguistic and non-linguistic response (e.g., producing a word while pointing to a prompt card). Response type is reported for both baseline and intervention; note that non-linguistic and combination responses were still possible in baseline, since participants could gesture or point to images in the book. *No responses* (i.e., failure to respond after a Level 4 prompt) were coded as such.

- Research question 4*: Median prompting level*

- The *prompting level* (*0–4*) at which the child responded accurately was recorded for each trial. Trials for which the child never responded or never responded accurately were not included in this analysis. The median score, as opposed to the mean, was chosen to describe the average level for each session because prompting level was an ordinal variable. Prompting level is only reported for intervention because baseline only included Level 0 prompts.

- Research question 5: *Responses to bids*

- *Percent response to secure attention prompts.* Each response to a secure attention prompt was coded as either *yes* or *no*, based on whether the child looked at or pointed to the image in the book that the interventionist identified. Responses are reported for both baseline and intervention.
- *Percent response to intentional pauses.* A response to an intentional pause was coded as either *yes* or *no*, based on whether the child produced an initiation (verbal or nonverbal). Responses are reported for both baseline and intervention.

### 2.8 Analysis

For each dependent variable, the impact of the independent variable was measured for each trial (i.e., question plus associated prompts) and summarized and plotted as a percentage or median response across each session, as described below. Each dependent variable was analyzed visually, following recommendations of Lobo et al. (2017). Within each phase (baseline and intervention), we examined each dependent variable’s level (i.e., mean or median), stability, and trend. We analyzed across-phase effects by assessing differences in level for baseline versus intervention, immediacy of the effect, and whether changes occurred only after each participant moved from baseline to intervention.

Intervention effectiveness was also evaluated quantitatively using Tau-U (Parker et al., 2011), a popular measure of non-overlap and effect size that also assesses statistical significance. Tau-U represents a family of measures that adjust for trends in baseline and intervention in different ways. Decisions about which Tau-U measure to use were based on recommendations from Fingerhut et al. (2021). First, for each individual and dependent variable, the baseline data were tested for the presence of a significant baseline trend. When there was no significant baseline trend, Tau-U_A vs. B_ (A vs. B phase non-overlap) was reported. When such a trend was present, Tau-U_Trend A_ (non-overlap with control for baseline trend) was reported for Dillan because he only had four baseline sessions, and Tau-U_Adj_ (non-overlap with the Theil-Sen adjustment of trend; Tarlow, 2017) was reported for the other participants. Tau-U is bounded between −1 and 1 can be interpreted similarly to a correlation coefficient. The scan package (Wilbert and Lüke, 2023) in R (R Core Team, 2024) was used to compute all statistics. We considered that the data established a functional relation between the IV and a DV if the visual and quantitative effects were replicated across at least three participants.

#### 2.8.1 Reliability and procedural integrity

Each session was scored live by the interventionist, as well as video and audio recorded. Based on the video recordings, a second rater independently coded the dependent variables for 25% of all baseline and intervention sessions for each participant. Because use of the visual prompt cards was evident from the videos, raters were not blinded to the phase. Interrater reliability was computed using Cohen’s kappa coefficient, which accounts for the possibility that the agreement could be due to chance. Reliability ranged from κ = 0.79 (response type) to *κ* = 0.89 (response level), which is considered substantial to almost perfect agreement, with the exception of secure attention prompts, which had moderate agreement (*κ* = 0.45) (see Landis and Koch, 1977).

Procedural integrity was not formally assessed. Compliance with procedures was established by having the two interventionists observe each other and provide feedback, in addition to occasional observations and feedback by the co-authors. As was evident from the data tracking sheets and video recordings, due to interventionist error, three participants received only two secure attention prompts in one of their sessions. For intentional pauses, four participants received 0–2 pauses across 1–3 sessions each, and two participants received a session of four pauses.

## 3 Results

### 3.1 Overview of sessions

The number of baseline sessions, intervention sessions, and absences were as follows: Dillan – 4, 16, 2; Hayley – 5, 16, 1; Ben – 7, 11, 4; Tucker – 7, 8, 7; Oliver – 10, 7, 5; Wally – 14, 8, 0. No adverse effects were observed for any of the participants.

### 3.2 Research question 1: responsiveness

**Figure 2:**
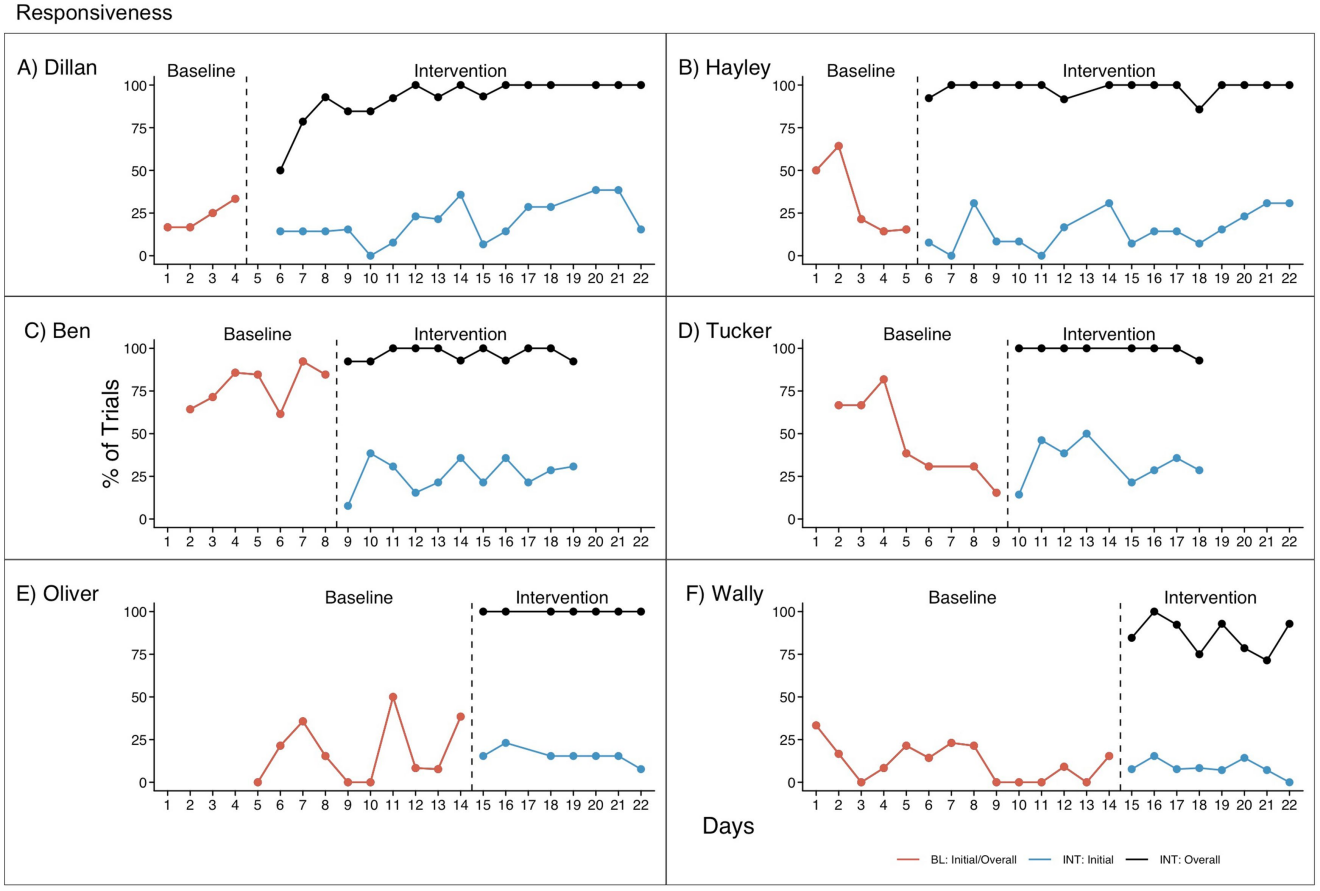
Responsiveness for **(A)** Dillan, **(B)** Hayley, **(C)** Ben, **(D)** Tucker, **(E)** Oliver, and **(F)** Wally. BL, Baseline. Initial and overall responses are equivalent in baseline since there was only one opportunity to respond. INT, Intervention. Initial responses represent (correct or incorrect) responses to the initial question/Level 0 prompt. Overall responses represent responses to a Level 0–4 prompt.

Responsiveness results are presented in Table 3 and Figure 2. When assessed individually, three participants (Dillan, Oliver, and Wally) showed no effect of intervention on initial responsiveness. The remaining three participants showed higher initial responsiveness in baseline than intervention, though this was significant only for Ben; this change for Ben occurred immediately after implementation of intervention. Hayley had non-significantly higher initial responsiveness in baseline with a non-significant decreasing trend. Tucker, on average, also had higher initial responsiveness in baseline; however, after correcting for his significant negative baseline trend, he showed significantly higher initial responsiveness in intervention. Given Hayley and Tucker’s negative trends in baseline, it is difficult to determine whether the decrease in initial responsiveness from baseline to intervention was due to intervention or other factors (e.g., familiarity/boredom with the study sessions). Additionally, there was not sufficient replication of these effects across participants to establish an impact of intervention on initial responsiveness.

**Table 3:** Responsiveness.

	Visual analysis					Quantitative analysis	
	Level change from BL to INT: Initial	Level change from BL to INT: Overall	BL stability and trend	INT stability and trend: initial	INT stability and trend: overall	Tau-U: initial	Tau-U: overall
Dillan	No	INT > BL	Stable positive trend	Unstable, no trend	Initial positive trend reaching ceiling	−0.31 (*p* = 0.34)	1 (*p* < 0.01)
Hayley	BL > INT	INT > BL	Negative trend	Unstable, no trend	Stable near ceiling	−0.52 (*p* = 0.09)	1 (*p* < 0.01)
Ben	BL > INT	INT > BL	Unstable, no trend	Unstable, no trend	Stable near ceiling	−1 (*p* < 0.01)	0.98 (*p* < 0.01)
Tucker	BL > INT^a^	INT > BL	Stable negative trend*	Unstable, no trend	Stable near ceiling	−0.38 (*p* = 0.22) 0.68 (*p* < 0.01)	1 (*p* < 0.01) 0.73 (*p* < 0.01)
Oliver	No	INT > BL	Unstable, no trend	Stable, no trend	Stable near ceiling	0.09 (*p* = 0.77)	1 (*p* < 0.01)
Wally	No	INT > BL	Unstable near floor	Stable near floor	Unstable near ceiling	−0.22 (*p* = 0.41)	1 (*p* < 0.01)

BL, Baseline. INT, Intervention. Initial, Initial responses. Overall, Overall responses. * = significant trend; if the trend was significant, uncorrected Tau-U is reported in roman font, and the appropriate baseline corrected Tau-U is reported in italics (see Data Analysis). When the significant negative baseline trend was corrected, Tucker had significantly higher initial responsiveness in intervention.

a Note that this is per visual assessment.

All participants showed significantly higher overall responses in intervention compared to baseline. (Note that initial responses in baseline are equivalent to overall responses because participants only had one opportunity to respond to each question in baseline). A similar effect can be observed when comparing initial to overall responses within the intervention phase, where the overall response rate was higher than the initial response rate. Since these effects were replicated more than three times (i.e., across all six participants), results suggest a functional relation between use of the RECALL prompting hierarchy and overall responsiveness. In addition, improvement of overall responses from baseline to intervention occurred immediately after each pair of participants was moved from baseline, showing good control for maturation and other threats to internal validity.

### 3.3 Research question 2: response accuracy

**Figure 3:**
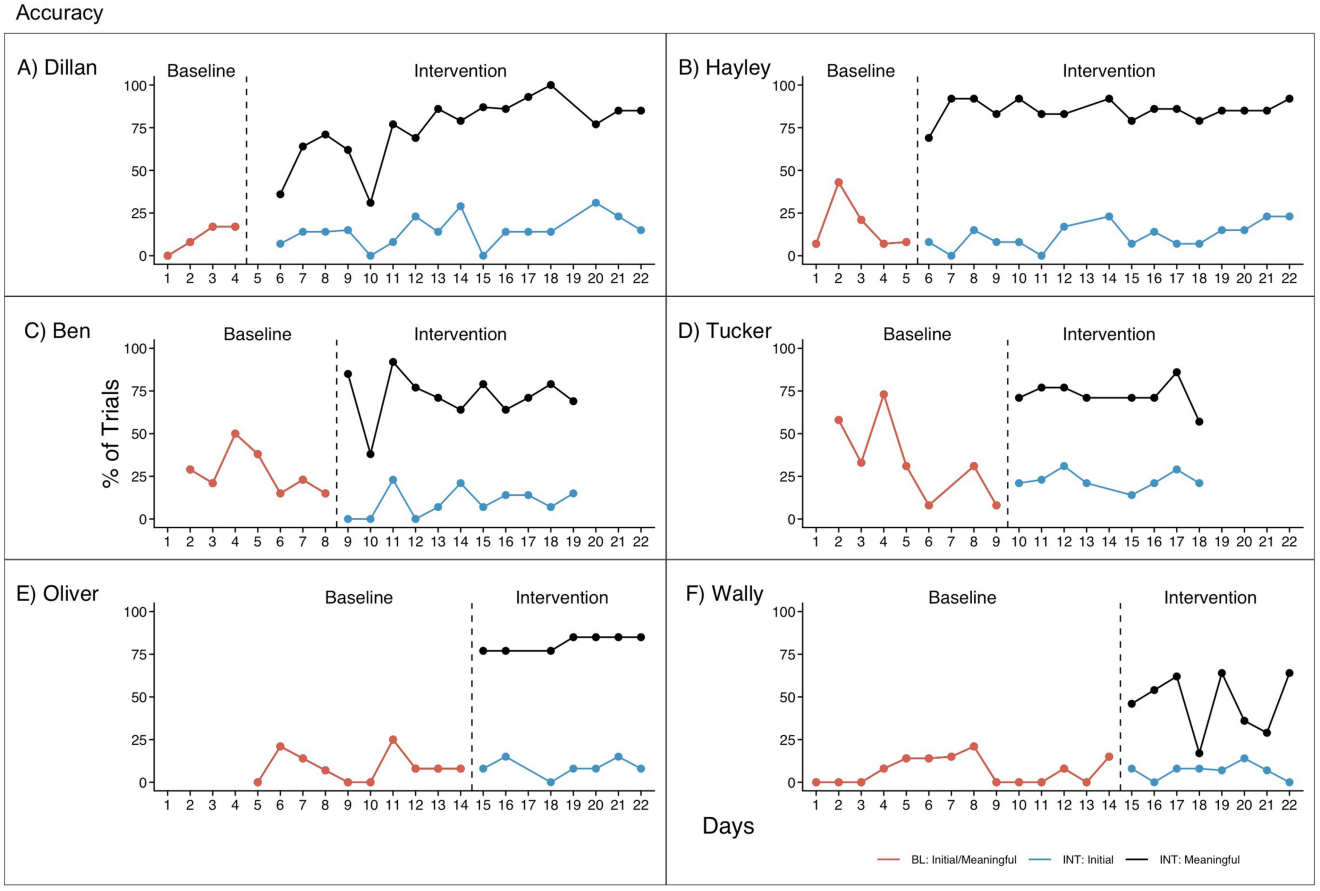
Response accuracy for **(A)** Dillan, **(B)** Hayley, **(C)** Ben, **(D)** Tucker, **(E)** Oliver, and **(F)** Wally. BL, Baseline. Initial and meaningful correct responses are equivalent in baseline since there was only one opportunity to respond. INT, Intervention. Initial correct responses represent correct responses to the initial question/Level 0 prompt. Meaningful correct responses represent correct responses to a Level 0–2 prompt, which required the child to demonstrate basic comprehension by selecting between at least two prompt cards.

Accuracy results are presented in Table 4 and Figure 3. Four participants showed no effect of intervention on initial correct responses. Ben had significantly – and immediately – lower initial accuracy in intervention than in baseline. Although Tucker showed no effect in visual analysis, after correcting for a significant negative trend in baseline, he had higher initial accuracy in intervention; however, this effect was not replicated across any other participants.

**Table 4:** Response Accuracy.

	Visual analysis					Quantitative analysis	
	Level change from BL to INT: Initial	Level change from BL to INT: Meaningful	BL stability and trend	INT stability and trend: initial	INT stability and trend: meaningful	Tau-U: Initial	Tau-U: meaningful
Dillan	No	INT > BL	Stable positive trend†	Unstable, no trend	Possible unstable positive trend	0.11 (*p* = 0.74)	1 (p < 0.01)
Hayley	No	INT > BL	Unstable, no trend	Unstable, no trend	Stable, no trend	−0.07 (*p* = 0.84)	1 (*p* < 0.01)
Ben	BL > INT	INT > BL	Unstable, no trend	Unstable, no trend	Unstable, no trend	−0.84 (*p* < 0.01)	0.97 (*p* < 0.01)
Tucker	No^a^	INT > BL	Unstable negative trend*	Stable, no trend	Stable, no trend	−0.40 (*p* = 0.20) 0.69 (*p* < 0.01)	0.79 (*p* < 0.05) 0.73 (*p* < 0.01)
Oliver	No	INT > BL	Unstable, no trend	Stable, no trend	Stable, no trend	0.14 (*p* = 0.66)	1 (*p* < 0.01)
Wally	No	INT > BL	Unstable near floor	Stable near floor	Unstable, no trend	−0.02 (*p* = 0.95)	0.98 (*p* < 0.01)

BL, Baseline. INT, Intervention. Initial, Initial correct responses. Meaningful, Meaningful correct responses. * = significant trend; if the trend was significant, uncorrected Tau-U is reported in roman font, and the appropriate baseline corrected Tau-U is reported in italics (see Data Analysis). † = marginal trend. When the significant negative baseline trend was corrected, Tucker had significantly higher initial accuracy in intervention.

a Note that this is per visual assessment.

All participants had significantly higher meaningful correct responses in intervention than in baseline. (Note that initial correct responses are equivalent to meaningful correct responses in baseline since participants only had one opportunity to respond to each question in baseline.) This effect appears to continue throughout intervention, where meaningful correct responses were notably higher than initial correct responses. Since these effects were replicated more than three times, this suggests a functional relation between use of the RECALL prompting hierarchy and meaningful correct responses. In addition, improvement of meaningful correct responses from baseline to intervention occurred immediately after switching phases for all pairs of participants, suggesting good experimental control for threats to internal validity.

### 3.4 Research question 3: response type

**Figure 4:**
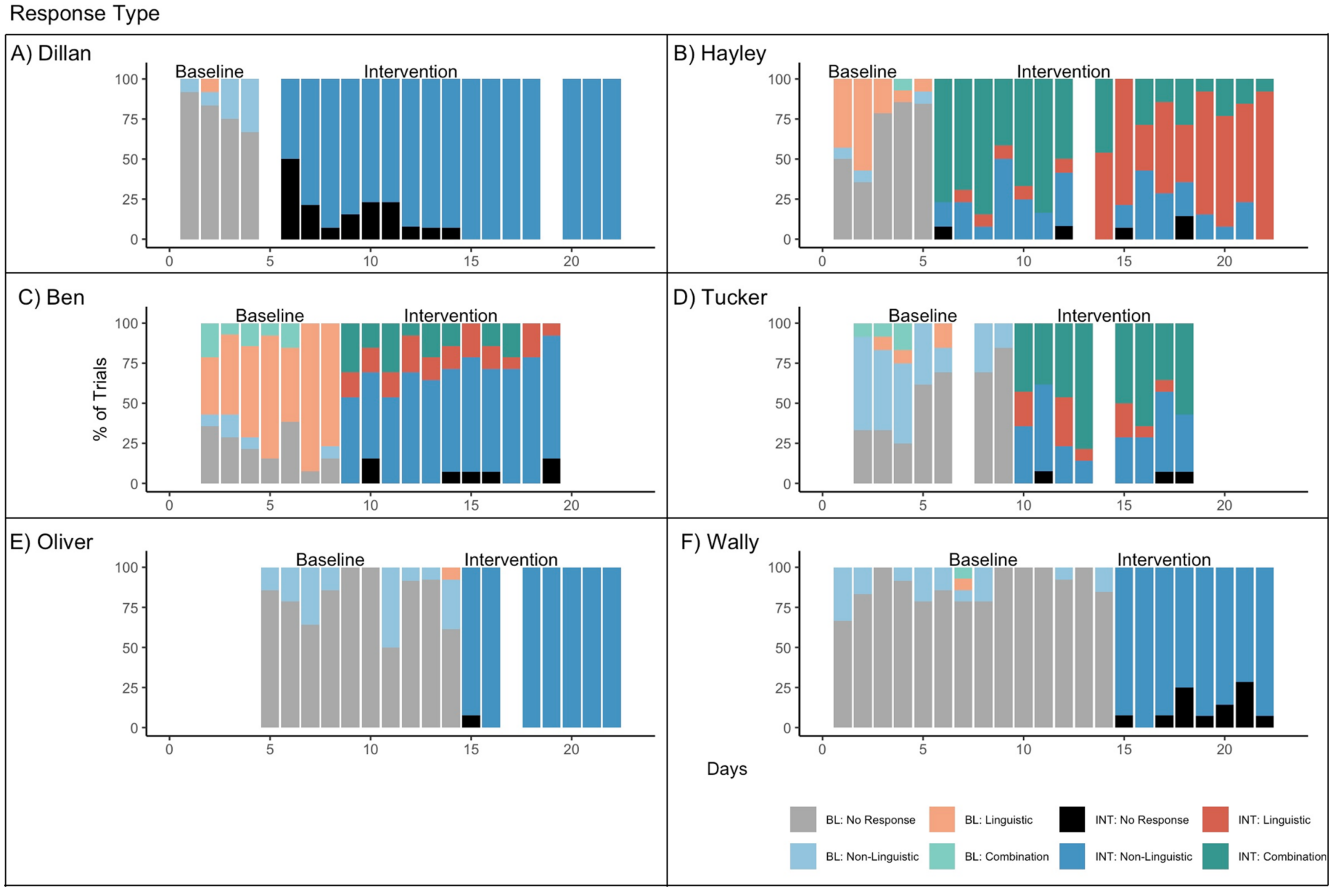
Response type for **(A)** Dillan, **(B)** Hayley, **(C)** Ben, **(D)** Tucker, **(E)** Oliver, and **(F)** Wally. BL = Baseline. INT = Intervention. No response reflects trials in which the child did not respond to the Level 0 prompt in baseline or to the Level 4 prompt in intervention. Non-linguistic responses included gestures, pointing, and picking up a prompt card. Linguistic responses included words, phrases, and signs. Combination responses were those that included both a linguistic and non-linguistic response element.

Response type results are reported in Figure 4. Because response type is a categorical variable, Tau-U is not reported. All participants decreased their number of no responses from baseline to intervention (Dillan: 79% baseline vs. 10% intervention, Hayley: 67% vs. 2%, Ben: 23% vs. 5%, Tucker: 54% vs. 3%, Oliver: 81% vs. 1%, Wally: 89% vs. 12%). For four participants, this corresponded to an increase in non-linguistic responses (Dillan 19% baseline vs. 90% intervention; Ben: 5% vs. 66%; Oliver: 18% vs. 99%; Wally: 10% vs. 88%). In addition to an increase in non-linguistic responses, Ben also showed a decrease in linguistic responses from baseline to intervention (62% vs. 15%). Hayley increased her use of all response types, including non-linguistic (4% vs. 20%), linguistic (27% vs. 37%; in particular see days 13–22) and combination (1% vs. 40%; in particular, see days 6–12). Finally, Tucker maintained a similar rate of non-linguistic responses in baseline and intervention (37% vs. 34%) but increased in his use of combination responses (5% vs. 52%). Visual analysis suggests that no participants changed response type until they moved from baseline to intervention, again suggesting good experimental control for threats to internal validity.

### 3.5 Research question 4: prompting level

Prompting level results are only reported for intervention sessions since baseline was limited to Level 0 prompts. Two participants showed no change in median prompting level required across intervention sessions (Hayley and Tucker were stable at Level 1). The remaining participants were more variable, as reflected by larger ranges of median prompting level across sessions (Dillan: median = 1, range = 1–4.5; Ben: median = 1, range = 1–3; Oliver: median = 2, range = 1–2; Wally: median = 2.5, range = 2–4). No trends across time were noted for any of the participants.

### 3.6 Research question 5: secure attention prompts and intentional pauses

**Figure 5:**
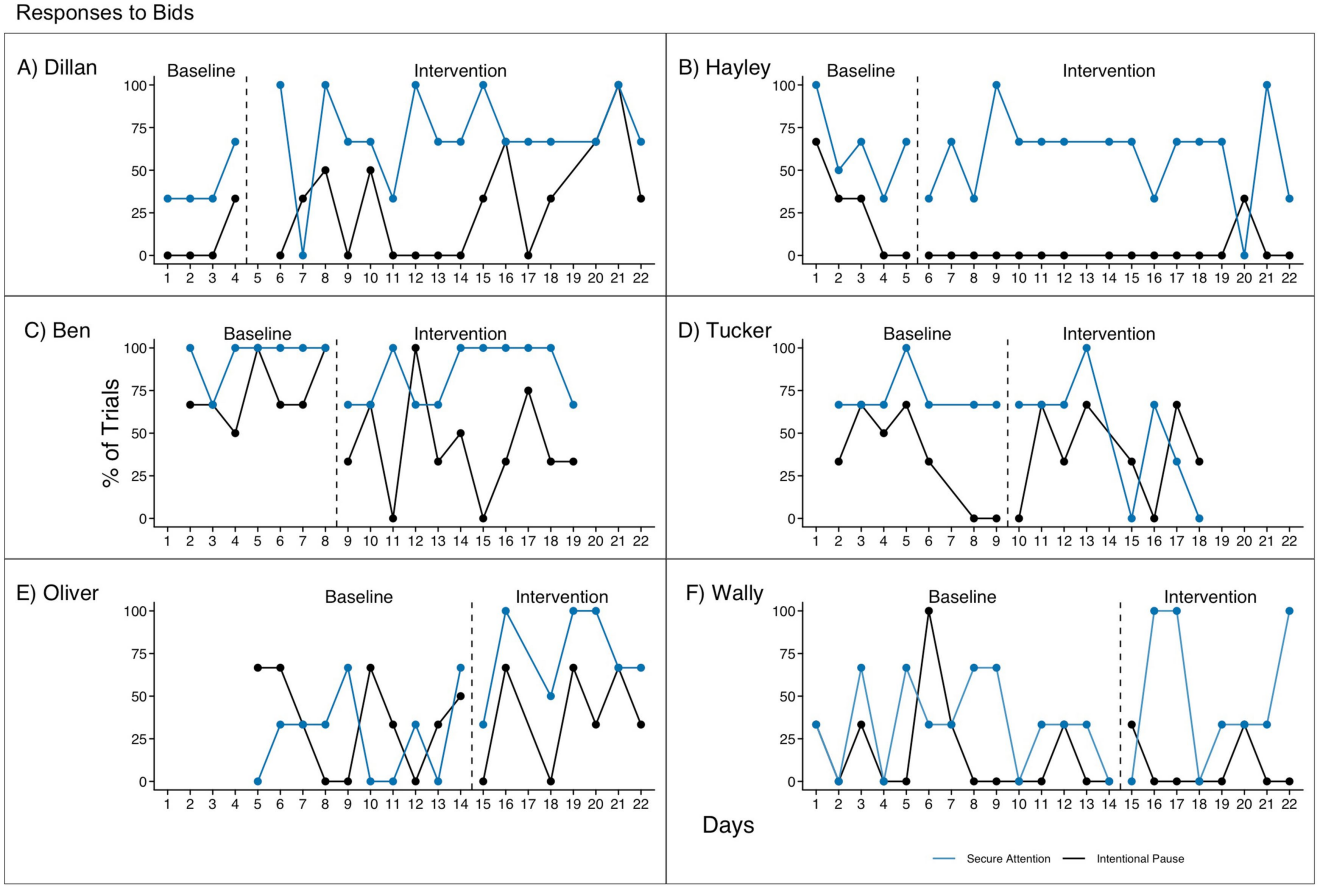
Responses to bids for **(A)** Dillan, **(B)** Hayley, **(C)** Ben, **(D)** Tucker, **(E)** Oliver, and **(F)** Wally. Responses to secure attention and intentional pause bids were coded as yes or no. Percentages are typically out of three trials.

Responses to secure attention prompts are reported in Table 5 and Figure 5, and responses to intentional pauses are reported in Table 6 and Figure 5. Substantial variability from session to session is seen in almost all children for both types of bids. Since there were typically only three bids of each type per session, this is not unexpected (e.g., responding to one additional bid would correspond to an increase of 33%).

**Table 5:** Secure attention prompts.

	Visual analysis			Quantitative analysis
	Level change from BL to INT: Initial	BL stability and trend^a^	INT stability and trend	Tau-U
Dillan	INT > BL	Stable, no trend	Unstable, no trend	0.72 (*p* = 0.05)
Hayley	No	Unstable, no trend	Unstable, no trend	−0.06 (*p* = 0.87)
Ben	No	Stable, no trend	Stable, no trend	−0.42 (*p* = 0.28)
Tucker	BL > INT	Stable, no trend	Unstable, possible negative trend	−0.44 (*p* = 0.27)
Oliver	INT > BL	Unstable, no trend	Unstable, no trend^b^	0.82 (*p* < 0.05)
Wally	No	Unstable, no trend	Unstable, no trend	0.22 (*p* = 0.45)

BL, Baseline. INT, Intervention.

a Given the small number of trials, the level was considered “stable” if the range was 33.3% or less.

b A trend is visually evident for Oliver when examining the entire time period.

**Table 6:** Intentional pauses.

	Visual analysis			Quantitative analysis
	Level change from BL to INT	BL stability and trend^a^	INT stability and trend	Tau-U
Dillan	INT > BL	Stable, no trend	Unstable, possible positive trend	0.49 (*p* = 0.24)
Hayley	BL > INT^b^	Stable negative trend*	Stable, mostly at floor	−0.69 (*p* = 0.07) 0.62 (*p* < 0.01)
Ben	BL > INT	Unstable, no trend	Unstable, no trend	−0.65 (*p* < 0.05)
Tucker	No	Unstable, no trend	Unstable, no trend	0.04 (*p* = 0.91)
Oliver	No	Unstable, no trend	Unstable, no trend	0.08 (*p* = 0.81)
Wally	No	Unstable, no trend	Stable, no trend	−0.17 (*p* = 0.63)

BL, Baseline. INT, Intervention. * = significant trend; if the trend was significant, uncorrected Tau-U is reported in roman font, and the appropriate baseline corrected Tau-U is reported in italics (see Data Analysis). When the significant negative baseline trend was corrected, Hayley was more responsive to intentional pauses in intervention.

a Given the small number of trials, the level was considered “stable” if the range was 33.3% or less.

b Note that this is per visual assessment.

Three of the participants showed no difference in responsiveness to secure attention prompts in baseline versus intervention or across time. One participant (Tucker) showed higher responsiveness to secure attention prompts in baseline, though this was not significant. The other two participants, Dillan and Oliver, were (marginally and significantly, respectively) more responsive to secure attention prompts in intervention. However, the timing of these effects with respect to the change from baseline to intervention is unclear, and we did not observe replications of the effect across other participants, so our results do not establish a functional relation between the RECALL prompting hierarchy and responses to secure attention bids. Instead, Dillan and Oliver’s improvements may be due to additional time, practice, or familiarity with the interventionist.

Three participants showed no change in responsiveness to intentional pauses from baseline to intervention. The other three participants showed different patterns. Dillan was more responsive to intentional pauses in intervention than in baseline, though the effect was not significant. Hayley was more responsive in baseline per visual analysis, though according to the quantitative analysis she was significantly more responsive in intervention after correcting for a significant negative trend in baseline. However, this effect should be interpreted with caution because her responses in intervention were nearly all at floor. Finally, Ben was significantly less responsive to intentional pauses in intervention than in baseline. As with secure attention prompts, without replications across participants or clear evidence of immediacy of the effects when switching from baseline to intervention, our results do not establish a functional relation between the RECALL prompting hierarchy and responses to intentional pause bids.

## 4 Discussion

The purpose of the present study was to investigate the impact of the RECALL prompting hierarchy (Whalon et al., 2015) on communicative responses in a sample of children with language delays/disorders, both with and without co-occurring autism.

### 4.1 Research questions 1–2: responsiveness and response accuracy

Overall responsiveness was consistently higher than initial responsiveness for all six participants. Thus, it seems that by providing more communicative opportunities via the RECALL prompting hierarchy, the intervention resulted in greater participant responsiveness. Further, the percentage of meaningful responses (responses for which the child had to select correctly between two or more options) was substantially higher for all six participants in the intervention phase, suggesting that the increased responses were of high quality. These changes coincided with the timing of the transition from baseline to intervention. These results are consistent with those reported by Towson et al. (2021) in their systematic review of shared interactive book reading interventions for children with disabilities, who reported large effect sizes for increased child responses across several reviewed studies. Our results are also consistent with Whalon et al.’s (2015) findings that incorrect and Level 3 prompts decreased and that Level 1–2 responses increased with RECALL and with Lo and Shum’s (2021) findings that responsiveness and engagement in reading increased from pre- to post-testing for participants who received the RECALL intervention in their randomized control trial. However, unlike Whalon et al. (2015), who reported an increase in initial correct responses (referred to as “spontaneous correct” or “unprompted correct responses”) for all four of their participants with RECALL, we did not see this effect. It is possible that this is due to the higher pre-existing language skills of Whalon et al.’s participants (PLS-5 scores of 67–72, vs. 50–60 for participants in the current study). Our study was also shorter in duration (22 vs. 67 sessions), so it is possible we would have seen improvement in initial correct responses had the intervention continued longer.

Future research should test if the benefits of the RECALL prompting hierarchy for responsiveness would be maintained if prompting cards were faded over time. Additionally, participant-specific reinforcers could be added to the intervention to motivate children to respond to initial questions, which may help with the transition away from prompting cards. For example, Whalon et al. (2015) found that one of their participant’s responsiveness to visuals increased once an individualized reinforcement system was introduced.

### 4.2 Research question 3: response types

All participants in the current study exhibited a change in their response types from baseline to intervention, substantially decreasing their percentage of no responses (i.e., an increase in overall responsiveness, as discussed above). For four of the participants, no responses tended to be replaced by non-linguistic responses, such as pointing. However, one participant (Ben) also replaced linguistic responses with non-linguistic responses when he moved from baseline to intervention, perhaps because it is easier to point than speak.

Prior to the start of the study, graduate clinicians working with Dillan, Oliver, and Wally described their primary mode of communication as non-linguistic (gestures and non-linguistic vocalizations). The addition of visual prompting cards provided these participants with a non-linguistic avenue to respond, thereby increasing their responsiveness and opportunities to engage in shared book reading. The other three participants primarily communicated linguistically with 1–4 word utterances. Ben showed a preference for non-linguistic communication when provided with prompt cards, while Hayley and Tucker increased their linguistic and/or combination responses. Thus, taken together, our results suggest that the visual prompt cards encouraged both non-linguistic and linguistic responses, depending on the participant and their typical mode of communication. Previous studies on RECALL did not measure response type, preventing a direct comparison of our results with theirs. However, our results are consistent with a recent meta-analysis by Boyle et al. (2019) who reported that shared reading interventions, broadly, led to positive effects in receptive and expressive language, as well as both communicative and non-communicative acts for participants on the autism spectrum. Future research should compare the impacts of a visual prompting hierarchy like RECALL to a verbal-only prompting hierarchy for children with different typical modes of communication. For example, previous studies have reported an increase in verbal communication using a verbal only least to most prompting hierarchy (e.g., vocal prompting only without visuals) during dialogic reading (Fleury and Schwartz, 2017; Queiroz et al., 2020). Future research should also examine how to best individualize prompts for children, depending on their current communication abilities.

### 4.3 Research question 4: prompting level

No participants in the current study demonstrated a decrease in the level of prompting required to produce a correct response across intervention sessions. Whalon et al. (2015) did not report on prompting level to the same extent as the current study, preventing a direct comparison of our findings. However, their data suggest that all participants still required a combination of different prompt levels at the end of the intervention. It is possible that a longer duration of intervention is necessary to see a decrease in prompting level, particularly given that participants may have come to expect and depend on the visual prompt cards as a potentially easier way to respond.

### 4.4 Research question 5: response to adult bids

Although RECALL was designed to elicit and improve joint attention skills in autistic children with autism, Whalon et al. (2015) did not directly report their participants’ responses to secure attention prompts or intentional pauses. In the current study, we measured responses to these two prompt types. The majority of participants did not show any changes in responsiveness to secure attention prompts or intentional pauses. A few participants were more or less responsive to these prompts in intervention than in baseline. Lower responsiveness in intervention (or in later sessions) could be due to habituation to the prompts. Higher responsiveness could be due to the intervention: while bids themselves were implemented the same way in baseline and intervention, the inclusion of the prompting hierarchy in intervention may have increased overall engagement for some participants. Alternatively, since the timing of the effects is not clearly related to the switch from baseline to intervention, it is possible that improvement was due to additional practice or improvement over time, perhaps due to increased comfort with the interventionist.

The purpose of the secure attention prompts and intentional pauses was, in part, to give the participants opportunities to initiate communication. However, our results suggest that these strategies may not be helpful for all children. In contrast to our findings, Whalon et al. (2015) reported that three of four of their participants, all autistic, increased the frequency of their spontaneous initiations, both verbal and nonverbal, though this was not tracked specifically in response to secure attention prompts or intentional pauses, across the course of the intervention. This discrepancy between our results and theirs could be due to the longer period of intervention used by Whalon et al. (2015) and/or the generally higher language skills of their participants.

The broader literature on interactive shared book reading interventions also reflects these difficulties and discrepancies in improving child initiations. In their systematic review, Towson et al. (2021) reported a range of different effect sizes across studies regarding their effectiveness in increasing child initiations, from no effect through large effects. It may be that children with language delays with or without co-occurring autism require more explicit training in responding to adult bids for securing attention and on how to initiate interactions, as well as more time working on the development of these skills, to see improvements. Developing these skills may help scaffold the development of joint attention so future studies on RECALL may also want to more directly measure joint attention (i.e., triadic engagement between the adult, child, and object; e.g., Mundy et al., 1990).

### 4.5 Limitations

There are several limitations in this study. First, the study was conducted at a preschool laboratory during a summer session, which limited the time frame of the study. The study lasted six weeks (22 days) with a one-week break in the middle, so there may not have been enough time to see improvement in some areas, especially for those who were last to switch from baseline to intervention and for difficult-to-target skills such as responding to adult bids. Relatedly, we did not assess the maintenance of intervention effects or the generalization of performance. These are important measurements to include in future research. Second, as with any single subject design study, the limited number of participants (*n* = 6) and study design prevent generalization to the larger and very diverse population of children with language delays and disorders, both with and without co-occurring autism. However, the sample size is consistent with other similar single subject studies, including Whalon et al. (2015) (*n* = 4) and Fleury and Schwartz (2017) (*n* = 9). Third, participants had little to no motivation to respond to the initial question (Level 0 prompt) once intervention started since participants had learned that response cards would soon follow the first question if they did not respond. As noted previously, future studies should add participant-specific reinforcements, as participants may be more motivated to respond during Level 0. Fourth, the current study did not examine participants’ responses to different question types (e.g., wh-inference versus emotion identification). Future research should examine participants’ responses to wh-inference and emotion identification questions specifically, as these questions are designed to aid individuals who struggle with social communication. Fifth, although the current study included children with language delays both with and without co-occurring autism, we did not examine differences between these two different types of participants. Although language delays are seen in both groups, there are phenotypic differences. Future studies should consider comparing the effect of RECALL on children with language delays/disorders and children with language delays and co-occurring autism. Lastly, we did not directly measure social validity in our study. As such, we are unable to report on the participants’, caregivers’, or teachers/therapists’ perceptions of the study outcomes, changes in participant performance, or the intervention itself.

### 4.6 Clinical applications

Reviews of interactive shared book reading interventions have suggested this is a promising intervention avenue for improving the language skills of children with disabilities, including those on the autism spectrum (e.g., Boyle et al., 2019; Towson et al., 2021). RECALL, specifically, may be of interest to teachers, speech-language pathologists, other practitioners, and parents of children with autism and/or language delays/disorders who are interested in increasing meaningful responses in their children/clients. The initial set-up and preparation of materials (i.e., RECALL-type questions and visual prompt cards) might be an initial barrier; however, once set up, practitioners can continue to use the same resources with several children or on a rotating basis. However, despite its promise in increasing responsiveness and the number of meaningful correct responses, there may also be situations in which RECALL may be inappropriate or unnecessary. For example, RECALL did not appear to improve the participants’ responses to adult bids or initiations, and it may need to be adapted into a verbal-only prompting procedure to be effective at increasing oral responsiveness for children who use spoken language.

## Data Availability

The raw data supporting the conclusions of this article will be made available by the authors, without undue reservation.
