# Supplementary material for: RECALL prompting hierarchy improves responsiveness for autistic children and children with language delay: a single-case design study
Source: Front Psychol. 2024 Oct 25;15:1435688. doi: 10.3389/fpsyg.2024.1435688 (PMC11545681; doi:10.3389/fpsyg.2024.1435688)
Supplement: Supplementary file 1 [file Table_1.pdf]

Supplemental Materials

List of Storybooks for Used for Baseline and Intervention

Dog's Colorful Day by Emma Dodd

Dodd E. (2001). *Dog's colorful day: a messy story about colors and counting* (First American). Dutton Children's Books.

The Day the Goose Got Loose by Reeve Lindbergh

Lindbergh R. & Kellogg S. (1995). *The day the goose got loose*. Puffin Pied Piper.

Pigs Aplenty, Pigs Galore! by David McPhail

McPhail D. (1993). *Pigs aplenty pigs galore!* (1st Puffin ed. 1996). Penguin Group.

The Snowy Day by Ezra Jack Keats

Keats E. J. (2011). *The snowy day* (50th anniversary). Viking Press.

The Summery Saturday Morning by Margaret Mahy

Mahy M. & Young S. (1998). *A summery Saturday morning*. Viking Press.

The Wolf's Chicken Stew by Keiko Kasza

Kasza K. (1987). *The wolf's chicken stew*. G.P. Putnam's Sons.

Sample Questions for One Session of *Dog's Colorful Day.*

Response options are in parentheses, with the correct response underlined when applicable.

Completion "Finish what I say…."
- Dog has a spot on his ______ (Ear/Tail/Nose).
- Now dog has six _____ (Spots/Bees/Clouds).

Recall
- What color is Dog's new spot? (Red/Blue/Purple)
- How did Dog get his purple spot? (Marker/Rain/Mud)

Open-ended
- What is happening in this picture? (Running/Sleeping/Swimming)
- What is he doing? (Getting in bed/Eating dinner/Going for a walk)

Wh-Questions
- Why did Dog take a bath? (Dirty/Hungry/Tired)
- What is brown on this page? (The Chocolate bar/Beach Ball/Grass).
- How many spots does Dog have now? (8/5/1)

Wh-Inference
- What do you think will happen next? (Eat/Walk/Sleep)

Distancing
- What type of ice cream do you like the best? (Chocolate/Vanilla/Strawberry)
- What type of juice do you like the best? (Orange/Apple/Grape)
- Which color do you like the best? (Pink/Blue/Green)

Emotion Identification
- How does Dog feel? (Tired/Sad/Happy)
