# Supplementary material for: RECALL prompting hierarchy improves responsiveness for autistic children and children with language delay: a single-case design study
Source: Front Psychol. 2024 Oct 25;15:1435688. doi: 10.3389/fpsyg.2024.1435688 (PMC11545681; doi:10.3389/fpsyg.2024.1435688)
Supplement: Supplementary file 2 [file Table_2.pdf]

Alternating Treatments Addendum to RECALL Prompting Hierarchy Improves Responsiveness for Children with Autism and Developmental Language Disorders: A Single-Case Design Study

1 The Alternating Treatments (ATD) Design

In addition to the six children tested using a multiple baselines design that are reported in the main manuscript, two additional children were tested using an alternating treatments design (ATD). We chose to test two participants using ATD because it allows researchers to quickly compare the effects of treatment and baseline, and it is more sensitive to rapid changes in behavior (Kadzin, 2011). However, ATD is not appropriate if there is a risk of carryover effects, in which the impact of intervention (e.g., learning) carries over to subsequent baseline sessions. This can obscure potential differences between baseline and intervention (see Discussion, below). Since some of the dependent variables measured in this study are at risk for carryover effects, we are reporting these results as a supplement rather than as part of the main manuscript.

2 Materials and Methods

The ATD participants were given the pseudonyms Zack and Matt. Zack was an Asian male aged 5;5 with a diagnosis of autism and language delay. His IQ score from the KBIT-2 was 103. His language standard scores from the PLS-5 (obtained 8 months after the study concluded) were as follows: 74 for auditory comprehension, 71 for expressive communication, 76 for total language. His vocabulary standard score from the PPVT-4 was 89. His graduate clinician reported that he primarily communicated using full sentences. He was assigned to interventionist 2. Zack was selected for ATD because he had limited availability due to a pre-scheduled family vacation.

Matt was a Caucasian male aged 4;5 with a diagnosis of autism and language delay, though the autism diagnosis was obtained after the study was completed. All of his standardized assessments were completed 8 months after the study concluded. His IQ score on the KBIT-2 was 42. His language standard scores from the PLS-5 were as follows: 69 for auditory comprehension, 56 for expressive communication, and 60 for total language. His vocabulary standard score from the PPVT-4 was 58. His graduate clinician reported that he primarily communicated with 1-word utterances, pointing, and gestures. He was assigned to interventionist 1. Matt was selected as the second ATD participant pseudo-randomly (because he chose the same book as Matt during the first baseline session).

Methods for the ATD study were the same as those reported in the main manuscript, with two design-specific exceptions. First, for ATD participants, the first session was always baseline, and the final week was always intervention. In between, a coin flip determined which condition each child participated in each day. Second, in addition to the analysis methods described in the main manuscript, we examined across-phase effects for the ATD participants by looking for separation between the plotted lines for baseline versus intervention.

3 Results

Zack had 8 baseline sessions, 9 intervention sessions, and 5 absences; Matt had 7, 15, and 0, respectively.

3.1 Research Question 1: Responsiveness

Neither Zack nor Matt showed an effect of intervention on initial responsiveness. Both showed significantly higher overall responses in intervention compared to baseline, though the effect was marginal for Zack due to near ceiling initial responsiveness in baseline. Responsiveness results for ATD are presented in Supplemental Figure 1 and Supplemental Table 1.

Supplemental Figure 1. Responsiveness for ATD. BL = Baseline. Initial and overall responses are equivalent in baseline since there was only one opportunity to respond. INT = Intervention. Initial responses represent (correct or incorrect) responses to the initial question/Level 0 prompt. Overall responses represent responses to a Level 0-4 prompt. The dashed vertical line indicates when the participant was switched to only intervention sessions.

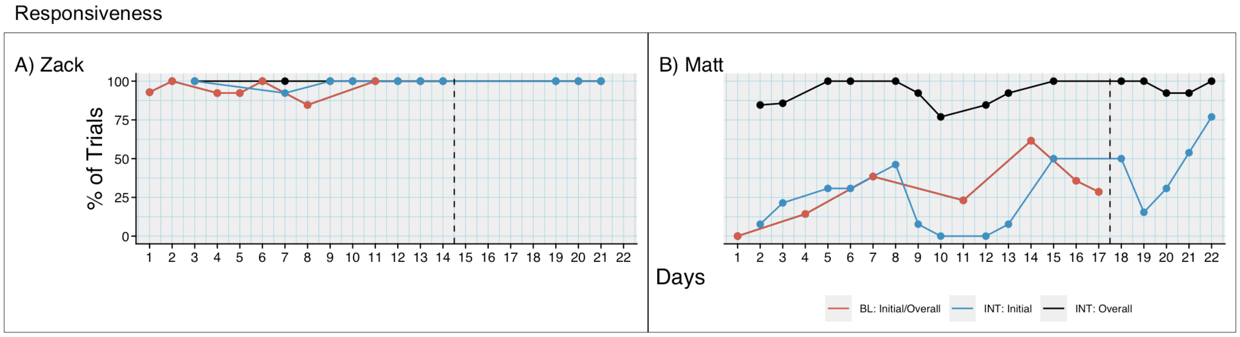

Supplemental Table 1. Responsiveness for ATD
BL = Baseline. INT= Intervention. Initial = Initial responses. Overall = Overall responses.

	Visual Analysis					Quantitative Analysis	
	**Level change from BL → INT: Initial**	**Level change from BL → INT: Overall**	**BL stability and trend**	**INT stability and trend: Initial**	**INT stability and trend: Overall**	**Tau-U: Initial**	**Tau-U: Overall**
Zack	No	No	Stable, no trend	Stable, no trend	Stable near ceiling	.51 (p = .18)	.67 (p = .08)
Matt	No	INT > BL	Unstable positive trend	Unstable, no trend	Unstable near ceiling	-.03 (p = .92)	1 (p < .01)

3.2 Research Question 2: Response Accuracy

Neither Zack nor Matt showed an effect of intervention on initial correct responses. Both showed significantly higher meaningful correct responses in intervention than in baseline. Accuracy results for ATD are presented in Supplemental Figure 2 and Supplemental Table 2.

Supplemental Figure 2. Response Accuracy for ATD. BL = Baseline. Initial and meaningful correct responses are equivalent in baseline since there was only one opportunity to respond. INT = Intervention. Initial correct responses represent correct responses to the initial question/Level 0 prompt. Meaningful correct responses represent correct responses to a Level 0-2 prompt, which required the child to demonstrate basic comprehension by selecting between at least two prompt cards. The dashed vertical line indicates when the participant was switched to only intervention sessions.

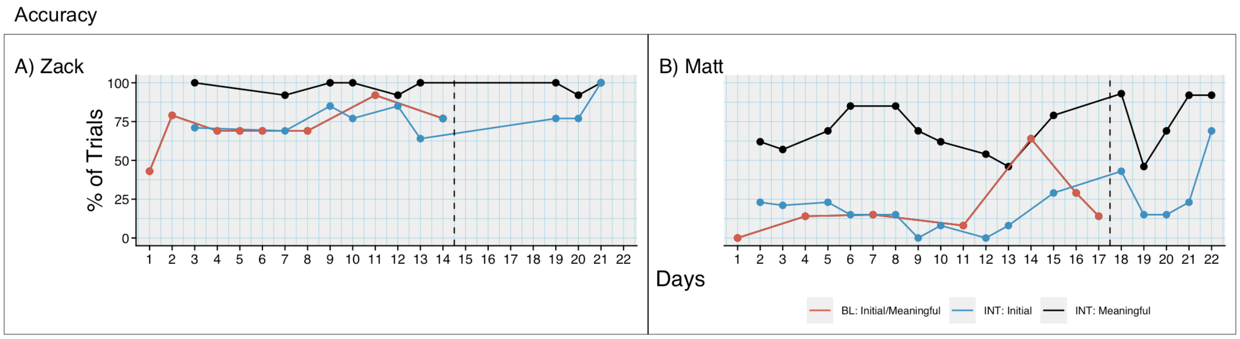

Supplemental Table 2. Response Accuracy for ATD
BL = Baseline. INT= Intervention. Initial = Initial correct responses. Meaningful = Meaningful correct responses.

	Visual Analysis					Quantitative Analysis	
	**Level change from BL → INT: Initial**	**Level change from BL → INT: Meaning-ful**	**BL stability and trend**	**INT stability and trend: Initial**	**INT stability and trend: Meaning-ful**	**Tau-U: Initial**	**Tau-U: Meaning -ful**
Zack	No	INT > BL	Stable, no trend	Stable, no trend	Stable, no trend	.34 (*p* = .27)	.92 (*p* < .01)
Matt	No	INT > BL	Unstable, no trend	Unstable, with possible positive trend at the end	Unstable, no trend	.16 (*p* = .56)	.89 (*p* < .01)

3.3 Research Question 3: Response Type

Zack used primarily linguistic responses in both baseline (82%) and intervention (83%). Matt decreased his percentage of no responses from baseline (72%) to intervention (8%), with a

corresponding increase in non-linguistic responses (26% baseline vs. 91% intervention). Response type results for ATD are presented in Supplemental Figure 3.

Supplemental Figure 3. Response Type. BL = Baseline. INT = Intervention. No response reflects trials in which the child did not respond to the Level 0 prompt in baseline or to the Level 4 prompt in intervention. Non-linguistic responses included gestures, pointing, and picking up a prompt card. Linguistic responses included words, phrases, and signs. Combination responses were those that included both a linguistic and non-linguistic response element.

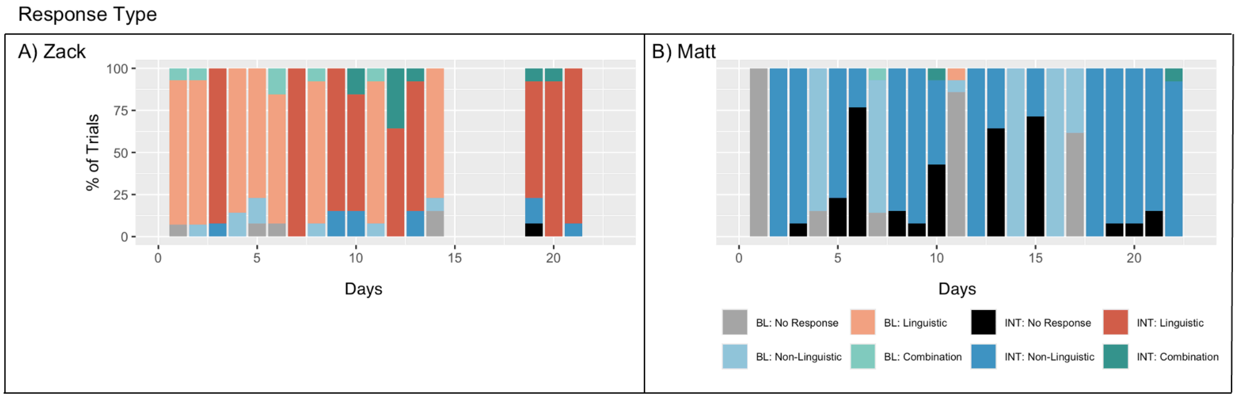

3.4 Research Question 4: Prompting Level

Zack typically responded to level 0 prompts in both baseline and intervention. Matt showed more variability, with a median prompting level of 1 and a range of 0-3. No trends across time were evident for either participant.

3.5 Research Question 5: Secure Attention Prompts and Intentional Pauses

Neither Zack nor Matt showed a difference in responsiveness to secure attention prompts or intentional pauses from baseline to intervention or across time (Supplemental Figure 4 and Supplemental Tables 3 and 4).

Supplemental Figure 4. Responses to Bids for ATD. BL = Baseline. INT = Intervention. Responses to secure attention and intentional pause bids were coded as yes or no. Percentages are typically out of three trials. The dashed vertical line indicates when the participant was switched to only intervention sessions.

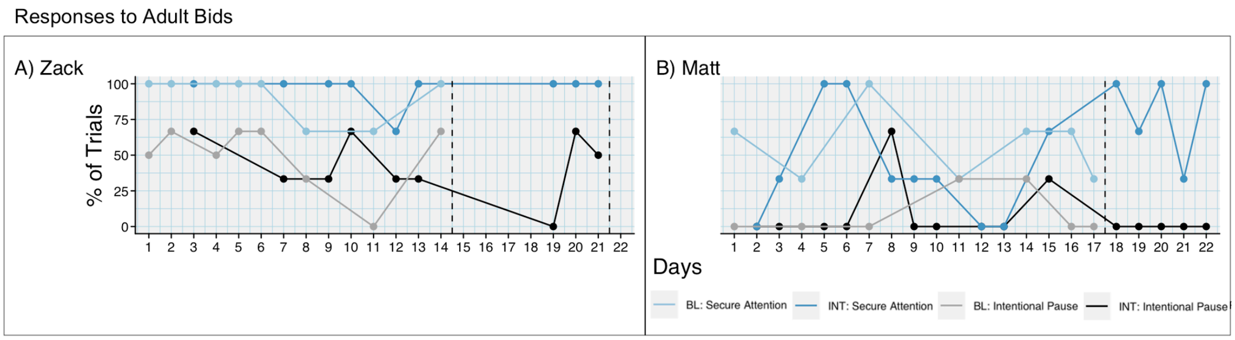

Supplemental Table 3. Secure Attention Prompts for ATD
BL = Baseline. INT= Intervention. [a]Given the small number of trials, the level was considered "stable" if the range was 33.3% or less.

	Visual Analysis			Quantitative Analysis
	**Level change from BL → INT**	**BL stability[a] and trend**	**INT stability and trend**	**Tau-U**
Zack	No	Stable near ceiling, no trend	Stable near ceiling, no trend	.21 (p = .63)
Matt	No	Unstable, no trend	Unstable, no trend	-.08 (p = .81)

Supplemental Table 4. Intentional Pauses for ATD
BL = Baseline. INT= Intervention. [a]Given the small number of trials, the level was considered "stable" if the range was 33.3% or less.

	Visual Analysis			Quantitative Analysis
	**Level change from BL → INT**	**BL stability[a] and trend**	**INT stability and trend**	**Tau-U**
Zack	No	Unstable, no trend	Unstable, no trend	-.27 (p = .41)
Matt	No	Stable, no trend	Stable with an outlier, no trend	-.20 (p = .62)

4 Discussion

Research Questions 1 and 2: Responsiveness and Response Accuracy. Findings related to responsiveness and correct responses are strengthened when considering ATD participants

alongside those reported in the main manuscript, who were tested using a multiple baselines across participants design. Including the RECALL prompting hierarchy (i.e., intervention) increased both overall responsiveness (though Zack already had a high response rate to the initial question, so he only showed marginal improvement) and percentage of meaningful correct responses for all participants across both designs, but not initial responsiveness or initial correct responses. Overall responsiveness and meaningful correct responses are not likely to be at high risk for carryover effects in ATD in the short term, since intervention allowed additional opportunities to respond and additional opportunities to produce a correct response that were not available in baseline. However, if the intervention is successful in the longer term, we would hope to see increased responsiveness and increased meaningful correct responses carry over to situations in which the prompting hierarchy is not used (i.e., baseline). Therefore, it is possible that the effects for participants in the ATD were underestimated due to carryover effects from intervention.

Research Question 3: Response Types. Use of the RECALL prompting hierarchy appeared to influence response type for seven out of eight total participants across the two designs, largely due to a decrease in no responses. Zack from ATD was the one exception to this, since he primarily responded linguistically throughout. It is possible that response type is vulnerable to carryover effects; for example, the use of visual prompt cards in intervention may encourage participants to point instead of answering verbally, which may carryover to baseline sessions in ATD (e.g., participants could respond by pointing to pictures in the book). However, this is not a concern for either Zack or Matt, since neither notably changed their percentage of linguistic responses from baseline to intervention.

Research Question 4: Prompting Level. Median prompting level was only relevant for intervention sessions, so carryover effects are not a concern. Like those participants reported in the main manuscript, neither ATD participant decreased the level of prompting required to produce a correct response across intervention sessions.

Research Question 5: Response to Adult Bids. We would expect carryover effects for any changes in responsiveness to adult bids, since the bids did not differ in baseline versus intervention and any improvement in responsiveness to secure attention prompts or intentional pauses would be likely to continue, regardless of experimental condition. Participants reported in the main manuscript showed variable performance on these two measures. In both cases, half (three out of six) of the multiple baselines participants showed no difference in responsiveness to either type of bid in baseline versus intervention or across time, with the other half showing variable patterns. The ATD participants showed no difference in responsiveness to either type of bid in baseline versus intervention or across time, further suggesting that RECALL may not be effective at improving responsiveness to adult bids, at least over the course of six weeks.
